# Human and Canine Blastomycosis Cases Associated with Riverside Neighborhood, Wisconsin, USA, December 2021–March 2022^1^

**DOI:** 10.3201/eid3012.240390

**Published:** 2024-12

**Authors:** Hannah E. Segaloff, Karen Wu, Samantha L. Williams, Summer Shaw, Shanna Miko, Lindsay A. Parnell, Andrew S. Hanzlicek, Kendra M. Carlson, Mark Lindsley, Ryan P. Westergaard, Mitsuru Toda, Suzanne N. Gibbons-Burgener

**Affiliations:** Centers for Disease Control and Prevention, Atlanta, Georgia, USA (H.E. Segaloff, K. Wu, S.L. Williams, S. Miko, L.A. Parnell, M. Lindsley, M. Toda); Wisconsin Department of Health Services, Madison, Wisconsin, USA (H.E Segaloff, S. Shaw, R.P. Westergaard, S.N. Gibbons-Burgener); MiraVista Diagnostics, Indianapolis, Indiana, USA (A. Hanzlicek, K.M. Carlson)

**Keywords:** zoonoses, fungi, blastomycosis, serology, disease outbreaks, Wisconsin, United States

## Abstract

We investigated a blastomycosis cluster among humans and canines in a neighborhood in Wisconsin, United States. We conducted interviews and collected serum specimens for *Blastomyces* antibody testing by enzyme immunoassay. Although no definitive exposure was identified, evidence supports potential exposures from the riverbank, riverside trails or yards, or construction dust.

Blastomycosis, caused by the dimorphic fungus *Blastomyces*, is a potentially severe disease of humans and animals. *Blastomyces* favors moist and organically rich soil, and soil disruption can lead to aerosolization and inhalation of infectious *Blastomyces* conidia (*1*,*2*). Most blastomycosis cases in the United States are sporadic and occur in the midwestern, south-central, and southeastern states. Infrequently, clusters of blastomycosis are reported because of common occupational and recreational activities near waterways (*3*).

In January 2022, St. Croix County Public Health, the Wisconsin Department of Health Services, and the Centers for Disease Control and Prevention (CDC) were notified of a cluster of human and canine blastomycosis cases near the Willow River in St. Croix County, Wisconsin (*4*). A clinical veterinarian reported 4 canine cases living within a radius of a few miles; 2 cases among persons living in the same area were reported to public health. The multiagency team investigated this cluster to identify risk factors for blastomycosis and to encourage persons in blastomycosis-endemic areas with consistent symptoms to seek healthcare.

The population at risk was defined as persons living within a 1.5-mile diameter of where the human and canine cases occurred. Members of the investigation team administered questionnaires to consenting households (Appendix 1), and collected information on exposures, symptoms, and risk factors for blastomycosis occurring during September 2021–March 2022.

Respondents were eligible to participate in serologic testing; pet owners were offered serologic testing for their dogs. We collected 5–10 mL of venous blood; serum samples were sent to MiraVista Diagnostics (Indianapolis, IN, USA) for *Blastomyces* spp. IgG testing by enzyme immunoassays (EIA) for human and canine serum samples and by immunodiffusion assay for human serum samples (*5*). This activity was reviewed by CDC and was conducted consistent with applicable federal law and CDC policy.

We defined human cases of blastomycosis among neighborhood residents who had illness onset during September 2021–March 2022. Included residents met clinical case criteria according to the Council of State and Territorial Epidemiologists case definition and met either confirmatory laboratory criteria or presumptive laboratory criteria, including a positive *Blastomyces* antigen test or a detection of serum antibody through immunodiffusion or EIA (*6*). Canine cases were dogs that exhibited clinical signs and either had received a diagnosis of blastomycosis from a veterinarian during September 2021–March 2022 or had detectable *Blastomyces* spp. antibodies.

We described the demographics, recreational exposure history, symptoms, and any symptom-related healthcare visits of persons by clinical case status. We compared recreational exposures between those with antibodies detected (positive or intermediate results) with exposures of those without antibodies detected. Prevalence ratios (PRs) and 95% CIs were calculated using robust Poisson models. All analyses were completed using R version 4.1.3 (The R Project for Statistical Computing, https://www.r-project.org).

We interviewed members of 60 households, 46 (77%) of whom participated in serologic testing (Figure). The median length of time lived in the neighborhood at time of interview was 4.9 years (range 1 month–56.5 years) (Table 1). The median distance to the in-neighborhood river was 342 (range 1.7–791.0) meters.

**Figure:**
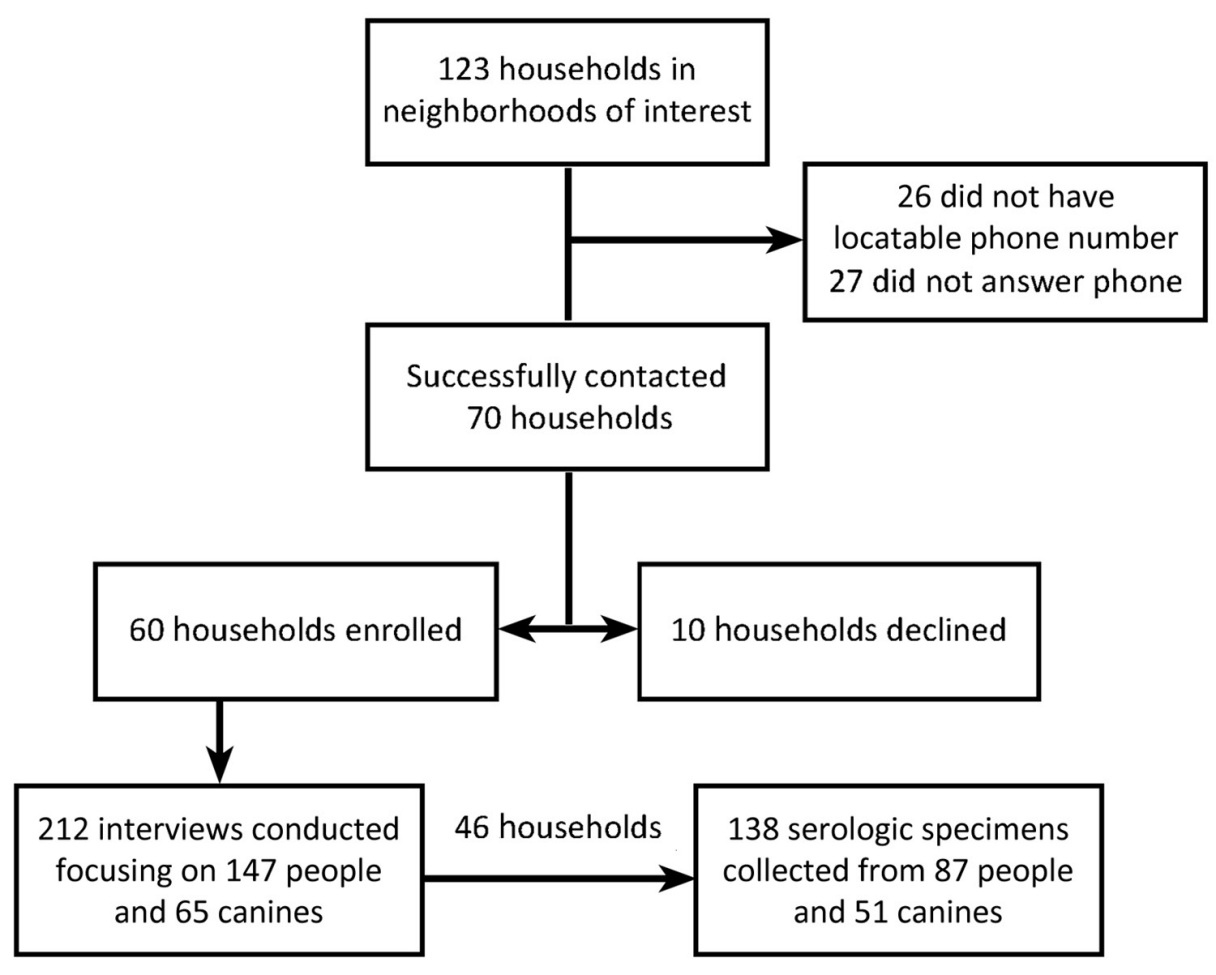
Flowchart of enrollment of households into investigation of human and canine blastomycosis cases associated with riverside neighborhood, Wisconsin, USA, December 2021–March 2022. Enrolled refers to a household in which >1 family member participated in the interview.

**Table 1:** Characteristics of 60 enrolled households during investigation of human and canine blastomycosis cases associated with riverside neighborhood, Wisconsin, USA, December 2021–March 2022*

Characteristic	Values
No. humans in household	2 (1–7) [2.0–3.0]
No. humans tested for antibodies in household	2 (0–4) [0.8–2.0]
No. humans with antibodies detected in household†	1 (0–3) [0–1.0]
No. dogs in household	1 (0–4) [0–1.3]
No. dogs tested for antibodies in household‡	1 (0–4) [0–1.0]
No. dogs with antibodies detected in household	0 (0–2) [0]
Length of time living in neighborhood	4.9 y (1 mo–56.5 y) [3.2 y–15.5 y]
Distance of house from construction, m	144 (0–814.8) [92.7–219.4]
Distance of house from river, m	341.7 (1.7–791.0) [231.1–463.2]
No. (%) households in neighborhood	
Neighborhood 1	20 (33.3)
Neighborhood 2	29 (48.3)
Neighborhood 3	11 (18.3)
No. (%) households with a clinically diagnosed case	
Human case	5 (8.3)
Canine case	6 (10.0)
Both human and canine cases	0 (0.0)
No. (%) households with any antibodies detected, n = 46	34 (73.9)
Humans with detected antibodies†	31 (67.4)
Canines with detected antibodies	7 (15.2)
Both humans and canines with detected antibodies‡	4 (6.7)
No. (%) household members (human or canine) with antibodies detected, n = 46	
0	12 (26.1)
1	22 (47.8)
2	7 (15.2)
3	4 (8.7)
4	1 (2.2)
No. (%) households with a clinically diagnosed case, n = 11	
Neighborhood 1, n = 20	2 (10.0)
Neighborhood 2, n = 29	8 (27.6)
Neighborhood 3, n = 11	1 (9.1)
No. (%) households that participated in the serosurvey, n = 46	
Neighborhood 1, n = 20	15 (75.0)
Neighborhood 2, n = 29	24 (82.8)
Neighborhood 3, n = 11	7 (63.6)
No. (%) households with antibodies detected, n = 46	
Neighborhood 1, n = 15	11 (73.3)
Neighborhood 2, n = 24	18 (75.0)
Neighborhood 3, n = 7	5 (71.4)

*Values are median (range) [IQR] except as indicated. IQR, interquartile range. †For humans, antibodies were considered detected in those returning a positive or intermediate result. ‡Antibody testing was completed by MiraVista Diagnostics for *Blastomyces* human and canine enzyme immunoassay.

During our investigation, 3 previously unreported human cases and 2 additional canine cases of blastomycosis were identified clinically after public health notification to local veterinarians, clinicians, and residents. A total of 5 human and 6 canine cases were included in this investigation (Appendix 2 Tables 1, 2).

The median age among human case-patients was 54 years, and most (60%, n = 3) had >1 comorbidity. All persons reported cough, fever, and fatigue associated with their illness. The initial 2 reported patients were hospitalized, and 1 died (*4*) (Appendix 2 Table 1). Isolates from the 2 initial cases were sent to the CDC Mycotic Diseases Branch laboratory for species identification, which determined the species to be *Blastomyces gilchristii*. In the canine cases, 5 of 6 (83%) were in sporting breeds; less than half of participating dogs were sporting breeds (n = 26, 42%). All dogs with a blastomycosis diagnosis were lethargic and anorexic; most had difficulty breathing (83%, n = 5) and cough and fever (67%, n = 4) (Appendix 2 Table 2). Human and canine case exposures varied.

We performed serologic testing on 89 (61%) of 147 human participants and 51 (79%) of 65 dogs (by EIA for dogs and immunodiffusion for humans). A total of 87 persons consented to further testing; their serum samples were later tested by a newly approved EIA. Among persons tested for antibodies, 1 person had detectable *Blastomyces* antibodies detected by immunodiffusion (also detected by EIA), and 44 (51%) persons had IgG detected by EIA (Table 2). Demographics and recreational exposures did not vary by antibody status, but male participants had 1.5 (95% CI 1.0–2.3) times the prevalence of antibodies than did female participants. Persons who reported a recreational activity involving the in-neighborhood river had 1.5 (95% CI 0.9–2.4) times greater prevalence of detectable antibodies, but was not statistically significant.

**Table 2:** Characteristics by antibody detection among humans during investigation of human and canine blastomycosis cases associated with riverside neighborhood, Wisconsin, USA, December 2021–March 2022*

Characteristic	Total, N = 87	Antibodies detected, N = 44	Antibodies not detected, N = 43	Prevalence ratio (95% CI)	p value
Median time lived in neighborhood, y **(**IQR)	5.0 (3.1–13.5)	5.0 (3.5–14.0)	5.2 (2.8–13.5)	1.00 (0.99–1.00)	0.98
Median distance from construction, m **(**IQR)†	138 (92–191)	136 (102–213)	139 (92–179)	1.00 (0.99–1.00)	0.98
Median distance from river, m (IQR)	338 (247–483)	313 (193–437)	350 (286–513)	0.99 (0.99–1.00)	0.08
Neighborhood of residence					
Neighborhood 1	31 (35.6)	16 (36.4)	15 (34.9)	1.1 (0.7–1.7)	0.81
Neighborhood 2	43 (49.4)	21 (47.7)	22 (51.2)	Referent	
Neighborhood 3	13 (14.9)	7 (15.9)	6 (14.0)	1.1 (0.6–2.0)	0.74
Age group, y					
<18	7 (8.0)	5 (11.4)	2 (4.7)	1.4 (0.7–2.8)	0.30
18–64	64 (73.6)	31 (70.5)	33 (76.7)	1.0 (0.6–1.7)	0.91
>64	16 (18.4)	8 (18.2)	8 (18.6)	Referent	
Sex					
M	38 (43.7)	24 (54.5)	14 (32.6)	1.5 (1.0–2.3)	0.04
F	49 (56.3)	20 (45.5)	29 (67.4)	Referent	
Race/ethnicity					
White, non-Hispanic	84 (96.6)	42 (95.5)	42 (97.7)	0.8 (0.3–1.7)	0.50
Other	3 (3.4)	2 (4.5)	1 (2.3)	Referent	
Immunosuppressive chronic conditions‡					
Any	20 (23.0)	8 (18.2)	12 (27.9)	0.7 (0.4–1.3)	0.32
Diabetes	3 (3.4)	0 (0.0)	3 (7.0)	NA	
Asthma	5 (5.7)	2 (4.5)	3 (7.0)	0.8 (0.3–2.3)	0.66
Other	12 (13.8)	6 (13.6)	6 (14.0)	1.0 (0.5–1.8)	0.97
Smoking history					
Current	3 (3.4)	2 (3.4)	1 (2.3)	Referent	
Former	16 (18.4)	8 (18.2)	8 (18.6)	0.8 (0.3–1.9)	0.55
Never	68 (78.2)	34 (77.3)	34 (79.1)	0.8 (0.3–1.7)	0.50
Any recreational activity§	74 (85.1)	37 (84.1)	37 (86.0)	0.9 (0.5–1.6)	0.79
Any recreational activity in neighborhood	44 (50.6)	20 (45.5)	24 (55.8)	0.8 (0.5–1.2)	0.34
Any recreational activity in river	10 (11.5)	7 (15.9)	3 (7.0)	1.5 (0.9–2.4)	0.10
Any soil-disturbing activity¶	81 (93.1)	42 (95.5)	39 (90.7)	1.6 (0.5–4.9)	0.45
Any soil-disturbing activity in neighborhood	76 (87.4)	38 (87.4)	38 (88.4)	0.9 (0.5–1.6)	0.77
Used walking trails	43 (49.4)	21 (47.7)	22 (51.2)	0.9 (0.6–1.4)	0.75

*Values are no. (%) except as indicated. Antibody testing was completed by MiraVista Diagnostics for *Blastomyces* human and canine enzyme immunoassay. IQR, interquartile range; NA, not applicable because model does not provide meaningful value. †Distance to construction is measured as the distance in meters from the house to the nearest lot with a construction permit in 2021. ‡Chronic and immunosuppressive conditions consisted of chronic obstructive pulmonary disease, diabetes, cancer, arthritis, organ transplant, long-term steroid treatment, asthma, and asplenia. §Recreational activities consisted of hunting, fishing, visiting a cabin, camping, hiking, biking, using an ATV, going to a park, and kayaking ¶Soil disturbing activities consisted of chopping wood, excavation, gardening, mulching, exposure through one’s occupation, exposure to construction, lawn care, and composting.

Among the 51 dogs tested, 8 (16%) had detectable antibodies, of which 6 (75%) had a clinical diagnosis. Higher number of years lived in the neighborhood was associated with decreased antibody prevalence (PR 0.32; 95% CI 0.1–0.9). Dogs that walked on the trails (PR 10.0; 95% CI 1.3–75.4), specifically those that walked off-leash (PR 6.8; 95% CI 2.0–23.9) had a higher prevalence of antibodies compared with dogs who did not (Table 3). Dogs who lived farther from the river had lower prevalence of antibodies (PR 0.98; 95% CI 0.97–0.99).

**Table 3:** Characteristics by antibody detection among canines in investigation of human and canine blastomycosis cases associated with riverside neighborhood, Wisconsin, USA, December 2021–March 2022

Characteristic	Total, N = 51	Antibodies detected, N = 8	Antibodies not detected, N = 43	Prevalence ratio (95% CI)	p value
Median time lived in the neighborhood, y **(**IQR)	3.5 (2.0–5.6)	3.0 (1.8–3.2)	4.0 (2.0–5.6)	0.32 (0.1–0.9)	0.03
Median distance lived from construction, m **(**IQR)†	150 (92–214)	170 (121–183)	139 (92–257)	0.99 (0.98–1.00)	0.13
Median distance lived from river, m **(**IQR)	349 (271–555)	293 (256–341)	364 (286–578)	0.98 (0.97–0.99)	0.02
Neighborhood of residence					
Neighborhood 1	24 (47.1)	2 (25.0)	22 (51.2)	0.3 (0.1–1.2)	0.08
Neighborhood 2	19 (37.3)	6 (75.0)	13 (30.2)	Referent	
Neighborhood 3	8 (15.7)	0 (0.0)	8 (18.6)	0.0 (0.0–0.0)	<0.0001
Age group, y‡					
<2	12 (24.5)	3 (37.5)	9 (22.0)	1.5 (0.4–6.2)	0.58
2–8	19 (38.8)	2 (25.0)	17 (41.5)	0.6 (0.1–3.4)	0.59
>8 y	18 (36.7)	3 (37.5)	15 (36.6)	Referent	
Sex					
M neutered	23 (45.1)	3 (37.5)	20 (46.5)	0.7 (0.2–2.4)	0.52
M intact	3 (5.9)	0 (0.0)	3 (7.0)	0.0 (0.0–0.0)	<0.001
F spayed	25 (49.0)	5 (62.5)	20 (46.5)	Referent	
F intact	0 (0.0)	0 (0.0)	0 (0.0)	NA	
Breed§					
Sporting breed pedigree¶	21 (32.0)	6 (75.0)	15 (35.7)	4.1 (0.9–18.5)	0.06
Other	29 (58.0)	2 (25.0)	27(64.3)	Referent	
Weight, pounds				1.2 (0.7–2.0)	0.44
Small (<25)	8 (15.7)	0 (0.0)	8 (18.6)		
Medium (25–49)	14 (27.5)	3 (37.5)	11 (25.6)		
Large (>50)	29 (56.9)	5 (62.5)	24 (55.8)		
Any recreational activity#					
Yes	32 (62.7)	4 (50.0)	28 (65.1)	0.6 (0.2–2.1)	0.41
No	19 (37.3)	4 (50.0)	15 (34.9)		
Any recreation in neighborhood					
Yes	13 (25.5)	3 (37.5)	10 (23.3)	1.8 (0.5–6.3)	0.39
No	38 (74.5)	5 (62.5)	33 (76.7)		
Any soil-disturbing activity**					
Yes	46 (90.2)	4 (50.0)	42 (97.7)	0.1 (0.0–0.3)	<0.001
No	5 (9.8)	4 (50.0)	1 (2.3)		
Any soil-disturbing activity in neighborhood					
Yes	40 (78.4)	4 (50.0)	36 (83.7)	0.3 (0.1–0.9)	0.04
No	11 (21.6)	4 (50.0)	7 (16.3)		
Used trails	21 (41.2)	7 (87.5)	14 (32.6)	10.0 (1.3–75.4)	0.03
Off-leash on trail	10 (19.6)	5 (62.5)	5 (11.6)	6.8 (2.0–23.9)	0.003
Off-leash out of yard††	20 (39.2)	6 (75.0)	14 (32.6)	1.1 (0.9–1.4)	0.50
Recreation in river	10 (19.6)	2 (25.0)	8 (18.6)	1.4 (0.3–5.8)	0.67
Frequent digger	24 (47.1)	5 (62.5)	19 (44.2)	1.9 (0.5–7.0)	0.35

*Values are no. (%) except as indicated. Antibody testing was completed by MiraVista Diagnostics for *Blastomyces* human and canine enzyme immunoassay (EIA). NA, not applicable because model does not provide meaningful value. †Distance to construction is measured as the distance in meters from the house to the nearest lot with a construction permit in 2021. ‡Information on age was missing for 2 dogs. §Breed information was missing for 1 dog. ¶Sporting breed category includes mixed-breed dogs. #Recreational activities consisted of hunting, fishing, visiting a cabin, camping, hiking, visiting parks, visiting a dog park, and kayaking. **Soil-disturbing activities consisted of exposure to lawn care, excavation, or gardening. ††Information about time spent off-leash out of the yard was missing for 1 dog.

We investigated a Wisconsin cluster of blastomycosis in which 5 human cases, resulting in 2 hospitalizations and 1 death, and 6 canine cases were identified. Using a multidisciplinary One Health approach to trigger notification and investigation of this cluster expanded both the investigative team and the outreach and effects among residents, pet owners, veterinarians, construction workers, and healthcare providers. The elevated awareness likely led to additional residents and dogs identified with blastomycosis illness and exposures.

The serological survey revealed nearly half of residents had *Blastomyces* antibodies detected by EIA. Although this result suggests widespread exposure, the length of time that *Blastomyces* antibodies remain detectable in the blood and the baseline antibody prevalence in the community are unknown (*5*,*7*). Although low sensitivity of immunodiffusion limits its use for detecting antibodies (*8*), this study used an EIA targeting the BAD-1 antigen with increased sensitivity.

Although we were unable to identify a definitive exposure as the likely cause of this cluster, several sites such as the riverbank, riverside trails or yards, or construction dust, could have plausibly been sources of *Blastomyces* spores. Although not statistically significant, antibody prevalence was increased in persons who reported participating in activities along the river, as did dogs who walked on in-neighborhood trails (Appendix 2 Tables 3, Figure). Extensive neighborhood construction and excavation during the exposure period might have increased risk for exposure because most recent home construction occurred close to the river. Construction and excavation have been implicated in previous clusters of blastomycosis (*9*–*11*).

## Conclusion

Prevention of blastomycosis is challenging. Recommendations for persons in blastomycosis-endemic areas include avoiding activities that disrupt dirt and leaf litter and wearing a well-fitted, high-quality facemask during those activities. Immunocompromised persons are at higher risk for severe blastomycosis and should avoid those activities. Risk for blastomycosis cannot be eliminated, and symptoms of blastomycosis can be indistinguishable from other illnesses. Increasing disease awareness is critical to improve early identification and treatment of patients.

Appendix 1 Questionnaires used in investigation of human and canine blastomycosis cases associated with riverside neighborhood, Wisconsin, USA, December 2021–March 2022

Appendix 2 Additional information about human and canine blastomycosis cases associated with riverside neighborhood, Wisconsin, USA, December 2021–March 2022
