# Supplementary material for: Human and Canine Blastomycosis Cases Associated with Riverside Neighborhood, Wisconsin, USA, December 2021–March 2022
Source: Emerg Infect Dis. 2024 Dec;30(12):2633–8. doi: 10.3201/eid3012.240390 (PMC11616636; doi:10.3201/eid3012.240390)
Supplement: Appendix 1 — Questionnaires used in investigation of human and canine blastomycosis cases associated with riverside neighborhood, Wisconsin, USA, December 2021–March 2022 [file 24-0390-Techapp-s1.pdf]

Article DOI: https://doi.org/10.3201/eid3012.240390

EID cannot ensure accessibility for supplementary materials supplied by authors. Readers who have difficulty accessing supplementary content should contact the authors for assistance.

Human and Canine Blastomycosis Cases Associated with Riverside Neighborhood, Wisconsin, USA, December 2021–March 2022

Appendix 1

Questionnaires used in investigation of human and canine blastomycosis cases associated with a riverside neighborhood in Wisconsin, USA, December 2021–March 2022, are shown on the following pages.

Human Investigation Form

record ID ______________________

Opt-out indicator
(check this box if an individual declines consent)
*consent should have been asked in the household enrollment form

☐ opt-out

Household ID ______________________

Demographics

Person ID ______________________
(Format should start with [household_id_h])

First Name ______________________

Last Name ______________________

Date of Birth ______________________

Sex
- ◯ Male
- ◯ Female
- ◯ Transgender (unspecified)
- ◯ Transgender (male to female)
- ◯ Transgender (female to male)

Are you currently pregnant?
- ◯ Yes
- ◯ No

Have you been pregnant since September 1, 2021
- ◯ Yes
- ◯ No

Street Address ______________________

City ______________________

Zip Code ______________________

County ______________________

What is a good phone number? ______________________

Second phone number (if applicable)	__________________________________
Occupation	__________________________________
Employer location	__________________________________
What is your ethnicity?	◯ Hispanic or Latino<br>◯ Not Hispanic or Latino<br>◯ Unknown<br>◯ Refused
Which of the following would you say is your race? Choose all that apply.	☐ White<br>☐ Black<br>☐ Native American/Native Alaskan<br>☐ Asian<br>☐ Native Hawaiian/Other Pacific Islander<br>☐ Other ______<br>☐ Unknown<br>☐ Refused
Who is being interviewed?	◯ Patient/Client<br>◯ Parent/Guardian<br>◯ Other
If other, specify	__________________________________

Interviewer Information

Date of Interview	__________________________________
Interviewer Name	◯ Andrea<br>◯ Dallas<br>◯ Hannah<br>◯ Karen<br>◯ Lindsay<br>◯ Lori<br>◯ Matt<br>◯ Other<br>◯ Shanna<br>◯ Staci<br>◯ Sue<br>◯ Summer<br>◯ Sam
Interviewer Organization or Agency	◯ CDC<br>◯ WI-DHS<br>◯ SCC<br>◯ Other
We may contact the client again about this investigation. If they refuse any future contact, indicate here	☐ Refuses future contact

EXPOSURE HISTORY

Did the client participate in any of the following recreational outdoor activities since September 1, 2021. For all yes answers, please provide months of participation and specific location information.

Hunting	◯ Yes ◯ No ◯ Unknown
During which months did the client hunt?	☐ September<br>☐ October<br>☐ November<br>☐ December
Where did the client hunt?	__________________________
Fishing from shore	◯ Yes ◯ No ◯ Unknown
During which months did the client fish?	☐ September<br>☐ October<br>☐ November<br>☐ December
Where did the client fish?	__________________________
Visiting a cabin	◯ Yes ◯ No ◯ Unknown
During which months did the client visit a cabin?	☐ September<br>☐ October<br>☐ November<br>☐ December
Where did the client visit a cabin?	__________________________
Camping	◯ Yes ◯ No ◯ Unknown
During which months did the client go camping?	☐ September<br>☐ October<br>☐ November<br>☐ December
Where did the client go camping?	__________________________
Hiking/cross country running	◯ Yes ◯ No ◯ Unknown
During which months did the client go hiking/cross country running?	☐ September<br>☐ October<br>☐ November<br>☐ December
Where did the client go hiking/cross country running?	__________________________

Trail biking	◯ Yes ◯ No ◯ Unknown
During which months did the client go trail biking?	☐ September<br>☐ October<br>☐ November<br>☐ December
Where did the client go trail biking?	________________________________
Using an ATV	◯ Yes ◯ No ◯ Unknown
During which months did the client use an ATV?	☐ September<br>☐ October<br>☐ November<br>☐ December
Where did the client use an ATV?	________________________________
Visiting parks	◯ Yes ◯ No ◯ Unknown
During which months did the client visit parks?	☐ September<br>☐ October<br>☐ November<br>☐ December
Where were the parks the client visited?	________________________________
Kayaking, canoeing, or tubing	◯ Yes ◯ No ◯ Unknown
During which months did the client go kayaking, canoeing, or tubing?	☐ September<br>☐ October<br>☐ November<br>☐ December
Where did the client go kayaking, canoeing, or tubing?	________________________________
Other activity (specify)	◯ Yes ◯ No ◯ Unknown
Specify activity	________________________________
During which months did the client participate in the outdoor recreational activity?	☐ September<br>☐ October<br>☐ November<br>☐ December
Where did the client participate in the other recreational activity?	________________________________

Was the client exposed to disturbed earth from any of the following activities since September 1, 2021. Provide activity months and specific location information for all yes responses.

Wood/brush cutting	◯ Yes ◯ No ◯ Unknown
During which months was the client exposed to disturbed earth from wood/brush cutting?	☐ September<br>☐ October<br>☐ November<br>☐ December
Where was the client exposed to disturbed earth from wood/brush cutting?	__________________________________
Excavation	◯ Yes ◯ No ◯ Unknown
During which months was the client exposed to disturbed earth from excavation?	☐ September<br>☐ October<br>☐ November<br>☐ December
Where was the client exposed to disturbed earth from excavation?	__________________________________
Gardening/landscaping	◯ Yes ◯ No ◯ Unknown
During which months was the client exposed to disturbed earth from gardening/landscaping?	☐ September<br>☐ October<br>☐ November<br>☐ December
Where was the client exposed to disturbed earth from gardening/landscaping?	__________________________________
Mulch exposure	◯ Yes ◯ No ◯ Unknown
During which months was the client exposed to disturbed earth from mulch exposure?	☐ September<br>☐ October<br>☐ November<br>☐ December
Where was the client exposed to disturbed earth from mulch exposure?	__________________________________
Occupational exposure	◯ Yes ◯ No ◯ Unknown
During which months was the client exposed to disturbed earth from an occupational exposure?	☐ September<br>☐ October<br>☐ November<br>☐ December
Where was the client exposed to disturbed earth from an occupational exposure?	__________________________________
Construction (road, structural)	◯ Yes ◯ No ◯ Unknown

During which months was the client exposed to disturbed earth from construction?	☐ September<br>☐ October<br>☐ November<br>☐ December
Where was the client exposed to disturbed earth from construction?	__________________________
Lawn care (raking, mowing)	◯ Yes ◯ No ◯ Unknown
During which months was the client exposed to disturbed earth from lawn care?	☐ September<br>☐ October<br>☐ November<br>☐ December
Where was the client exposed to disturbed earth from lawn care?	__________________________
Composting	◯ Yes ◯ No ◯ Unknown
During which months was the client exposed to disturbed earth from composting?	☐ September<br>☐ October<br>☐ November<br>☐ December
Where was the client exposed to disturbed earth from composting?	__________________________
Other activity (specify)	◯ Yes ◯ No ◯ Unknown
Specify activity	__________________________
During which months was the client exposed to disturbed earth from this activity?	☐ September<br>☐ October<br>☐ November<br>☐ December
Where was the client exposed to disturbed earth from this activity?	__________________________
Did the client travel (in-state or out-of-state) since September 1, 2021?	◯ Yes<br>◯ No<br>◯ Unknown
If yes, when/where?	__________________________
When/where (2nd trip)	__________________________
How close does the client live to the Willow River? (Can ask client to look this up on a map)	◯ < =100 feet<br>◯ < =1/4 mile and >100 feet<br>◯ < = 1 mile and >1/4 mile<br>◯ Greater than 1 mile

In what year did the client move into their home?

____

In what month did the client move into their home?

- ◯ January
- ◯ February
- ◯ March
- ◯ April
- ◯ May
- ◯ June
- ◯ July
- ◯ August
- ◯ September
- ◯ October
- ◯ November
- ◯ December
- ◯ Don't know

Since September 1, 2021, has the client walked or ran along the in-neighborhood Willow River trail and conservancy area?

- ◯ Yes
- ◯ No
- ◯ Don't know

In which of these paths does the client use to enter and exit the trail and conservancy area?

	Enter	Exit
Southwest path off 150th Ave near turnabout (closest to 95th street and Glen View neighborhood)	☐	☐
West path off 96th street	☐	☐
Northcentral path off 154th Ave (runs N-S in a ravine between 9 backyards and has stairs)	☐	☐
Northeast path off 152nd Ave (east-most path with multi branches, closest to 100th St, has stairs)	☐	☐
Other path; describe ______	☐	☐

How frequently did they walk along or use the trail during the following months of 2021?

	May-Aug	Sept	Oct	Nov	December
Daily (5 or more days per week)	☐	☐	☐	☐	☐
Multiple times per week (2-4 days)	☐	☐	☐	☐	☐
Weekly (once per week)	☐	☐	☐	☐	☐
Multiple times a month (less than weekly)	☐	☐	☐	☐	☐
Once a month	☐	☐	☐	☐	☐
Never	☐	☐	☐	☐	☐

During the following months of 2021, did they use the trail for these special events or activities?

	May-Aug	Sept	Oct	Nov	Dec
Special event or gathering on the trail or in conservancy areas (Describe) ______	☐	☐	☐	☐	☐
Maintenance or upkeep of paths, trails or conservancy area (Describe) ______	☐	☐	☐	☐	☐
Other activity (Specify) ______	☐	☐	☐	☐	☐

Since September 1, 2021, has the client gone in or close to the Willow River near the neighborhood trail and conservancy area?

◯ Yes
◯ No
◯ Don't Know

Describe activity and month

____

Since September 1, 2021, has the client walked by or been near ongoing excavation or construction in the neighborhood?

◯ Yes
◯ No
◯ Don't Know

Thinking about the time from September 1 through December, on average, how many hours a week did the client spend outdoors, not including for their job?

	>10 hours	5 - 10 hours	3 - 5 hours	1-3 hours	< 1 hour
Sept and Oct	◯	◯	◯	◯	◯
Nov and Dec	◯	◯	◯	◯	◯

Notes

____

Risk Factors

Does the client have any of the following chronic/immunosuppressive medical conditions? *if other immunosuppressive medication is used, indicate in the 'other' option

☐ COPD (chronic obstructive pulmonary disease)
☐ Diabetes
☐ Cancer
☐ Rheumatoid arthritis
☐ Organ transplant
☐ Steroid treatment (long-term)
☐ Asthma
☐ Asplenia (absence of spleen- e.g., surgical removal)
☐ None
☐ Other ______

Is the client a smoker or has the client ever smoked? (including but not limited to cigarettes, cigars, pipe)

◯ Current smoker (or quit in the last 6 months)
◯ Previous smoker
◯ Never smoked

For how many years have they (or did they) smoke?

____

Quantity smoked per day (i.e. number of packs or cigars)?	______________________
Does or has the client used electronic cigarettes or vapor pens?	◯ Current daily user<br>◯ Current less than daily user<br>◯ Previous user<br>◯ Never user
Has anyone in the house ever been diagnosed with blastomycosis?	◯ Yes<br>◯ No<br>◯ Don't know
Who/When	______________________
Has anyone you know recently been diagnosed with blastomycosis?	◯ Yes<br>◯ No<br>◯ Don't know
Who/when	______________________
Does client or household have dogs?	◯ Yes<br>◯ No
How many dogs?	______________________
Does client or household have cats?	◯ Yes<br>◯ No
How many cats do they have that go outside?	______________________
Has the client ever owned a dog that was diagnosed with blastomycosis?	◯ Yes<br>◯ No<br>◯ Never owned a dog
When was the diagnosis made?	______________________
What is the veterinarian's name?	______________________
What is the veterinarian's phone number?	______________________

Past Clinical Illness?

Interviewer only (don't ask client): Is client a known blastomycosis case?
- ◯ Yes
- ◯ No

Since September 1, 2021, have you been diagnosed with community acquired pneumonia?
- ◯ Yes
- ◯ No

What symptoms did you experience?
- ☐ Cough
- ☐ Coughing up blood
- ☐ Shortness of breath
- ☐ Fever
- ☐ Chills
- ☐ Night sweats
- ☐ Weight loss
- ☐ Poor appetite
- ☐ Headache
- ☐ Fatigue
- ☐ Chest pain
- ☐ Back pain
- ☐ Single skin lesion
- ☐ Multiple skin lesion
- ☐ Other ______

When did you start showing symptoms ______

____

For how long were you sick? (Answer in days)

____

Where you prescribed any medication, such as an antibiotic?
- ◯ Yes
- ◯ No

Since September 1 2021, Have you had a respiratory illness with no known cause?
- ◯ Yes
- ◯ No

What symptoms did you experience?
- ☐ Cough
- ☐ Coughing up blood
- ☐ Shortness of breath
- ☐ Fever
- ☐ Chills
- ☐ Night sweats
- ☐ Weight loss
- ☐ Poor appetite
- ☐ Headache
- ☐ Fatigue
- ☐ Chest pain
- ☐ Back pain
- ☐ Single skin lesion
- ☐ Multiple skin lesion
- ☐ Other ______

When did you start showing symptoms? ______

____

For how long were you sick? (Days)

____

Where you prescribed a medication, such as an antibiotic?	◯ Yes<br>◯ No<br>◯ Unknown
Have you ever been diagnosed with blastomycosis?	◯ Yes<br>◯ No
What symptoms did you experience?	☐ Cough<br>☐ Coughing up blood<br>☐ Shortness of breath<br>☐ Fever<br>☐ Chills<br>☐ Night sweats<br>☐ Weight loss<br>☐ Poor appetite<br>☐ Headache<br>☐ Fatigue<br>☐ Chest pain<br>☐ Back pain<br>☐ Single skin lesion<br>☐ Multiple skin lesion<br>☐ Other ______
When did your doctor test you for blastomycosis? ______	______________________________
When did you start showing symptoms? ______	______________________________
For how long were you sick? (days)	______________________________
Did you doctor prescribe an anti-fungal medication (e.g. itraconazole or Sporanox)?	◯ Yes<br>◯ No<br>◯ Unknown
The fungus that causes blasto lives in previously undisturbed, moist, organically rich soil, often near the banks of waterways. Activities that disrupt the soil could allow the spores of the fungus to become airborne. Infection could occur when these spores are inhaled. Where do you think you were infected?	______________________________

____

Pet Investigation Form

record ID ______________________

Investigation Household ID: ______________________

Pet ID ______________________
(should start with [household_id_a])

Please ensure that human investigation form is complete for all persons living in the home

Demographic Information

Owner's Name ______________________

Street Address ______________________

City ______________________

State ______________________

County ______________________

Zip ______________________

Telephone ______________________

Pet's name ______________________

Pet's weight (lbs) ______________________

Species
- ◯ Dog
- ◯ Cat

Breed ______________________

Age (years) ______________________

Sex
- ◯ Male
- ◯ Female

Spayed/neutered	◯ Yes<br>◯ No
When was pet adopted or acquired?	__________________________________
Pet is primarily	◯ Indoors<br>◯ Outdoors<br>◯ Both
Veterinary Clinic or Veterinarian	__________________________________
Clinic Phone	__________________________________

Interviewer Information

Date of Interview	__________________________________
Interviewer Name	◯ Andrea<br>◯ Dallas<br>◯ Hannah<br>◯ Karen<br>◯ Lindsay<br>◯ Lori<br>◯ Matt<br>◯ Other<br>◯ Shanna<br>◯ Staci<br>◯ Sue<br>◯ Summer<br>◯ Sam
Agency or Organization	◯ CDC<br>◯ WI-DHS<br>◯ SCC<br>◯ Other
We may contact the client again about this investigation. If they refuse any future contact, indicate here.	◯ Refuses future contact
Since September 1, 2021, has the pet been sick? (if no, skip to Pet's Blastomycosis History)	◯ Yes<br>◯ No<br>◯ Unknown

if yes, please list new symptoms:

	Yes	No	Unknown
Cough	◯	◯	◯
Coughing up blood	◯	◯	◯
Difficulty breathing	◯	◯	◯
Skin bumps or sores	◯	◯	◯
Lameness/limping	◯	◯	◯
Fever	◯	◯	◯
Seizures	◯	◯	◯
Eye or vision problems	◯	◯	◯
Lethargy	◯	◯	◯
Poor appetite	◯	◯	◯
Weight loss	◯	◯	◯
Other symptoms ______	◯	◯	◯

When did the pet start showing symptoms? ______________________________

Did the pet recover or return to usual health?
◯ Yes
◯ No
◯ Unknown

For how long was the pet sick? ______________________________

Did the pet receive veterinary care?
◯ Yes
◯ No
◯ Unknown

If yes, when? ______________________________

Notes ______________________________________

Pet's Blastomycosis History

Was the pet ever diagnosed with blastomycosis?
◯ Yes
◯ No
◯ Unknown

When was pet diagnosed? ______________________________

Has the pet recovered?
◯ Yes
◯ No
◯ Unknown

Was the pet hospitalized for blastomycosis?
◯ Yes
◯ No
◯ Unknown

If so, where?

____

Was the pet treated for blastomycosis?

◯ Yes
◯ No
◯ Unknown

Risk Factors

Does the pet have any underlying health issues?

◯ Yes
◯ No
◯ Unknown

If yes, please describe:

____

Do you own any other animals?

◯ Yes
◯ No
◯ Unknown

Please fill out additional Animal Blastomycosis Investigation forms for other animals

If yes, have any of these other animals ever been diagnosed with blastomycosis?

◯ Yes
◯ No
◯ Unknown

If yes, when?

____

Has any person in the home ever been diagnosed with blastomycosis?

◯ Yes
◯ No
◯ Unknown

If yes, when?

____

Did the pet travel away from home since September 1, 2021?

◯ Yes
◯ No
◯ Unknown

if yes, where?

____

Does the pet live on or near a lake, river, stream, creek, or wetland?

◯ Yes
◯ No
◯ Unknown

if yes, what is the name of the body of water?

____

If yes, how far away is it?

◯ less than 100 ft
◯ less than 1/4 mile
◯ less than a mile
◯ greater than a mile

Does your pet routinely dig or disturb soil?	◯ Yes<br>◯ No<br>◯ Unknown
if yes, when/where (eg. in the yard, garden, during hikes)?	__________________________
Is your pet routinely allowed off leash outdoors?	◯ Yes<br>◯ No<br>◯ Unknown
if yes, when/where (eg. dog parks, during hikes)?	__________________________

EXPOSURE HISTORY

Did the dog participate in any of the following recreational outdoor activities since September 1, 2021. For all yes answers, please provide months of participation and specific locations.

Hunting	◯ Yes ◯ No ◯ Unknown
During which months did the dog participate in hunting?	☐ September<br>☐ October<br>☐ November<br>☐ December
Where did the dog participate in hunting?	__________________________
Fishing from shore	◯ Yes ◯ No ◯ Unknown
During which months did the dog participate in fishing?	☐ September<br>☐ October<br>☐ November<br>☐ December
Where did the dog participate in fishing?	__________________________
Visiting a cabin	◯ Yes ◯ No ◯ Unknown
During which months did the dog visit a cabin?	☐ September<br>☐ October<br>☐ November<br>☐ December
Where did the dog visit a cabin?	__________________________
Camping	◯ Yes ◯ No ◯ Unknown
During which months did the dog go camping?	☐ September<br>☐ October<br>☐ November<br>☐ December

Where did the dog go camping?	__________________________
Hiking/running	◯ Yes ◯ No ◯ Unknown
During which months did the dog participate in hiking/running?	☐ September<br>☐ October<br>☐ November<br>☐ December
Where did the dog participate in hiking/running?	__________________________
Visiting parks	◯ Yes ◯ No ◯ Unknown
During which months did the dog visit parks?	☐ September<br>☐ October<br>☐ November<br>☐ December
Where were the parks the dog visited?	__________________________
Visiting dog parks	◯ Yes ◯ No ◯ Unknown
During which months did the dog visit dog parks?	☐ September<br>☐ October<br>☐ November<br>☐ December
Where were the dog parks the dog visited?	__________________________
Kayaking, canoeing, or tubing	◯ Yes ◯ No ◯ Unknown
During which months did the dog participate in kayaking, canoeing, or tubing?	☐ September<br>☐ October<br>☐ November<br>☐ December
Where did the dog participate in kayaking, canoeing, or tubing?	__________________________
Other activity (specify)	◯ Yes ◯ No ◯ Unknown
Specify activity	__________________________
During which months did the dog participate in the activity?	☐ September<br>☐ October<br>☐ November<br>☐ December
Where did the dog participate in the activity?	__________________________

Was the pet exposed to disturbed earth or present for any of the following activities since September 1, 2021? Provide activity months and specific location information for all yes responses.

Lawn care (exposure to lawn refuse, present for raking, mowing)	◯ Yes ◯ No ◯ Unknown
During which months was the dog exposed to disturbed earth from lawn care?	☐ September<br>☐ October<br>☐ November<br>☐ December
Where was the dog exposed to disturbed earth from lawn care?	__________________
Excavation/construction (road building, underground utilities)	◯ Yes ◯ No ◯ Unknown
During which months was the dog exposed to disturbed earth from excavation/construction?	☐ September<br>☐ October<br>☐ November<br>☐ December
Where was the dog exposed to disturbed earth from excavation/construction?	__________________
Gardening/landscaping (including exposure to mulch or compost)	◯ Yes ◯ No ◯ Unknown
During which months was the dog exposed to disturbed earth from gardening/landscaping?	☐ September<br>☐ October<br>☐ November<br>☐ December
Where was the dog exposed to disturbed earth from gardening/landscaping?	__________________
Other activity (specify)	◯ Yes ◯ No ◯ Unknown
Specify	__________________
During which months was the dog exposed to disturbed earth from the activity?	☐ September<br>☐ October<br>☐ November<br>☐ December
Where was the dog exposed to disturbed earth from the activity?	__________________

Exposure History-Current Investigation Supplement

How close does the pet live to the Willow River? (likely same as owner and can determine on map)

- ◯ less than 100 ft
- ◯ less than 1/4 mile
- ◯ less than a mile
- ◯ greater than a mile

Since September 1, 2021, has the pet walked along the in-neighborhood Willow River trail and conservancy area?

- ◯ Yes
- ◯ No

If yes, which of these paths do they use to enter and exit the trail and conservancy area? Path may run behind backyard

	Enter	Exit
Southwest path off 150th Ave near turnabout (closest to 95th and Glen View neighborhood	☐	☐
West path off 96th Ave	☐	☐
Northcentral path off 154th Ave (runs N-S between 9 backyards in ravine, has stairs)	☐	☐
Northeast path off 152nd Ave (east-most path with multiple branches, closest to 100th St, has stiars)	☐	☐
Other path; Describe ______	☐	☐

if yes, how frequently did they walk along or use the trail during the following months of 2021?

	May-Aug	Sept	Oct	Nov	Dec
Daily (5 or more days)	☐	☐	☐	☐	☐
Multiple times per week (2-4 days)	☐	☐	☐	☐	☐
Once weekly	☐	☐	☐	☐	☐
Multiple times a month	☐	☐	☐	☐	☐
Once a month	☐	☐	☐	☐	☐
Never	☐	☐	☐	☐	☐

Describe conservancy sections used:

____

Is the pet allowed off-leash along the trail or in the conservancy area?

- ◯ Yes
- ◯ No

Since September 1, 2021, has the pet gone in or close to the Willow river near the neighborhood trail and conservancy area? (eg. swimming, wading, playing, exploring, marking)

- ◯ Yes
- ◯ No

if yes, describe activity and month

____

notes:

____

Since September 1, 2021, has your pet walked by or been near ongoing excavation or construction in the neighborhood?

◯ Yes
◯ No

notes

____

Thinking about the time from September 1 through December, on average, how many hours a week did your pet spend outdoors, including in their home yard?

	Greater than 10 hours	5-10 hours	3-5 hours	1-3 hours	Less than 1 hour
Sept & Oct	◯	◯	◯	◯	◯
Nov & Dec	◯	◯	◯	◯	◯

Complete only if pet is known to have blastomycosis diagnosis, (skip if they have not been diagnosed)

The fungus that causes blasto lives in previously undisturbed, moist organically rich soil, often near the banks of waterways. Activities that disrupt the soil could allow the spores of the fungus to become airborne. Infection could occur when these spore are inhaled. Where do you think your pet was infected with Blastomyces?

____

Other comments on outdoor activities

____

Investigation notes and details

____
