# Supplementary material for: Human and Canine Blastomycosis Cases Associated with Riverside Neighborhood, Wisconsin, USA, December 2021–March 2022
Source: Emerg Infect Dis. 2024 Dec;30(12):2633–8. doi: 10.3201/eid3012.240390 (PMC11616636; doi:10.3201/eid3012.240390)
Supplement: Appendix 2 — Additional information about human and canine blastomycosis cases associated with riverside neighborhood, Wisconsin, USA, December 2021–March 2022 [file 24-0390-Techapp-s2.pdf]

Article DOI: https://doi.org/10.3201/eid3012.240390

EID cannot ensure accessibility for supplementary materials supplied by authors. Readers who have difficulty accessing supplementary content should contact the authors for assistance.

Human and Canine Blastomycosis Cases Associated with Riverside Neighborhood, Wisconsin, USA, December 2021–March 2022

Appendix 2

Methods

Determination of Construction

Construction permits issued in 2021 in the neighborhood for septic or well installation, driveway pouring, and new home, garage, deck, pool, or basement building were obtained. Lots with a construction permit issued in 2021 were deemed under construction.

GIS Mapping and Distance Calculations

The addresses of lots were geocoded using the Melissa address locator (rooftop level) API via the WI-DHS Enterprise Geocoding Service (EGS). Spatial analyses were performed in ArcMap 10.7.0 (ESRI 2018). One hundred and sixteen of 133 lots were successfully geocoded to the house rooftop level. The remaining 17 homes were geocoded to the lot centroid by hand. Distance from each lot centroid or roof location to the nearest in-neighborhood body of water and to the nearest lot deemed under construction were measured using the Near tool in the Analysis toolbox in ArcMap 10.7.0. Geographic relation of the house to both the in-neighborhood river and houses under construction was depicted by graphing the distance from the water on the y-axis and the distance from the nearest home under construction on the x-axis. Median Euclidean distance from the nearest home and nearest construction was calculated and

compared between homes where antibodies were detected in a dog or person and homes where antibodies were not detected.

Results

Construction in the Neighborhoods of Interest and Proximity to the River

Of the 123 households identified in the neighborhoods' 131 lots, 19 had a construction permit in 2021. Median distance between houses under construction in 2021 and the in-neighborhood river was 111 m (IQR: 60, 387) compared to 351 m (IQR: 256, 484) for houses that did not have a construction permit (p = 0.001) (Appendix 2 Table 3). Among the 46 homes which had at least one person or dog tested for antibodies, the median distance to the river did not vary by antibody detection (Appendix 2 Figure, panels A, B; p = 0.30) nor did the median distance to construction (Appendix 2 Figure, panels A, C; p = 0.44). In addition, the Euclidean distance from the river and nearest home under construction did not vary by antibody presence (Appendix 2 Figure, panels A, D; p = 0.17).

Appendix 2 Table 1. Characteristics of confirmed or probable and suspected human blastomycosis cases during investigation of blastomycosis cluster, Wisconsin, 2021–2022*

Characteristic	Total, n = 147	Cases, n = 5	Suspected cases, n = 11	Non-Cases, n = 131
Median length of time lived in neighborhood, y (IQR)	5.2 y (3.2 y–14.5 y)	4.2 y (8 mo–15.8 y)	5.2 y (3.1 y–20.9 y)	5.2 y (3.2 y–13.8 y)
Median distance lived from construction, m (IQR)†	156 (99–214)	118 (113–357)	214 (184–808)	150 (97–208)
Median distance lived from river, m (IQR)	423 (298–590)	304 (117–377)	341 (313–578)	350 (241–483)
Median age, y (IQR)	42 (21–58)	54 (17–56)	39 (28–59.5)	43 (22–58)
Neighborhood of residence				
Neighborhood 1	58 (39.5)	1 (20.0)	1 (9.1)	56 (42.7)
Neighborhood 2	66 (44.9)	3 (60.0)	6 (54.5)	57 (43.5)
Neighborhood 3	23 (15.6)	1 (20.0)	4 (36.4)	18 (13.7)
Age group, y‡				
<18	30 (20.7)	2 (40.0)	2 (18.2)	26 (20.2)
18–64	95 (65.5)	2 (40.0)	6 (54.5)	87 (67.4)
>64	20 (13.8)	1 (20.0)	3 (27.3)	16 (12.4)
Sex				
M	67 (45.6)	4 (80.0)	4 (36.4)	59 (45.0)
F	80 (54.4)	1 (20.0)	7 (63.6)	72 (55.0)
Race				
White, non-Hispanic	142 (96.6)	5 (100.0)	10 (90.9)	127 (96.9)
Other	5 (3.4)	0 (0.0)	1 (9.1)	4 (3.1)
Any immunosuppressive medical condition§	30 (20.4)	3 (60.0)	2 (18.2)	25 (19.1)
Any recreational activity¶	110 (74.8)	4 (80.0)	10 (90.9)	96 (73.3)
Any recreational activity in neighborhood	72 (49.0)	4 (80.0)	5 (45.5)	63 (48.1)
Any recreational activity in river	40 (27.2)	3 (60.0)	3 (27.3)	34 (26.0)
Any soil-disturbing activity#	129 (87.8)	4 (80.0)	9 (81.8)	116 (88.5)
Any soil-disturbing activity in neighborhood	119 (81.0)	4 (80.0)	9 (81.8)	106 (80.9)
Used walking trails	60 (40.8)	3 (60.0)	5 (45.5)	52 (39.7)
Symptoms				
Any	28 (19.0)	5 (100)	11 (100)	12 (9.2)
Cough	26 (17.7)	5 (100)	11 (100)	11 (8.4)
Fatigue	15 (10.2)	5 (100)	10 (90.9)	1 (0.8)

Characteristic	Total, n = 147	Cases, n = 5	Suspected cases, n = 11	Non-Cases, n = 131
Fever	8 (5.4)	5 (100)	4 (36.4)	0 (0.0)
Chills	7 (4.8)	3 (60.0)	5 (45.5)	0 (0.0)
Shortness of Breath	7 (4.8)	3 (60.0)	3 (27.3)	2 (1.5)
Night Sweats	3 (2.0)	3 (60.0)	0 (0.0)	1 (0.8)
Poor appetite	3 (2.0)	1 (20.0)	2 (18.2)	0 (0.0)
Chest or back pain	3 (2.0)	1 (20.0)	1 (9.1)	1 (0.8)
Other	12 (8.2)	2 (40.0)	2 (18.2)	9 (6.9)
Hospitalized	2 (1.4)	2 (40.0)	0 (0.0)	0 (0.0)
Died	1 (0.7)	1 (20.0)	0 (0.0)	0 (0.0)

*Values are no. (%) except as indicated. Cases refer to confirmed or probable cases. Confirmed human cases were defined as people with clinically compatible illness who had confirmatory laboratory criteria. Probable cases included those with positive antibody testing and clinically compatible illness.
†Distance to construction is measured as the distance in meters from the house to the nearest lot with a construction permit in 2021.
‡Two people are missing information on age.
§Chronic and immunosuppressive conditions included COPD, diabetes, cancer, arthritis, organ transplant, long-term steroid treatment, asthma, and asplenia.
¶Recreational activities included hunting, fishing, visiting a cabin, camping, hiking, biking, using an ATV, going to a park, and kayaking.
#Soil-disturbing activities included chopping wood, excavation, gardening, mulching, exposure through one's occupation, exposure to construction, lawn care and composting.

Appendix 2 Table 2. Characteristics of clinical canine blastomycosis cases during investigation of blastomycosis cluster, Wisconsin, 2021–2022*

Characteristic	Total, n = 65	Cases, n = 6	Non-Cases, n = 59
Median time lived in the neighborhood, y (IQR)†	3.8 (2.0–5.6)	2.0 (1.5–3.2)	4.0 (2.0–5.7)
Median distance lived from construction, m (IQR)‡	154 (94–257)	155 (84–172)	155 (95–257)
Median distance lived from river, m (IQR)	351 (286–555)	319 (267–347)	364 (286–555)
Neighborhood of residence			
Neighborhood 1	31 (47.7)	1 (16.7)	30 (50.8)
Neighborhood 2	22 (33.8)	5 (83.3)	17 (28.8)
Neighborhood 3	12 (18.5)	0 (0.0)	12 (20.3)
Age group, y§			
<2	14 (23.0)	3 (50.0)	11 (20.0)
2–8	25 (41.0)	0 (0.0)	25 (45.5)
>8	22 (36.1)	3 (50.0)	19 (34.5)
Sex			
M neutered	26 (40.0)	1 (16.7)	25 (42.4)
M intact	4 (6.2)	0 (0.0)	4 (6.8)
F spayed	33 (50.8)	5 (83.3)	28 (47.5)
F intact	0 (0.0)	0 (0.0)	0 (0.0)
F (unknown)	2 (3.1)	0 (0.0)	2 (3.4)
Breed¶			
Sporting breed pedigree	26 (41.3)	5 (83.3)	22 (36.8)
Other	37 (58.7)	1 (16.7)	36 (63.2)
Weight#			
Small (<25 pounds)	10 (15.9)	0 (0.0)	10 (17.5)
Medium (25–49 pounds)	16 (25.4)	3 (50.0)	13 (22.8)
Large (≥50 pounds)	37 (58.7)	3 (50.0)	34 (59.6)
Symptoms			
Any	10 (15.4)	6 (100.0)	4 (6.8)
Cough	5 (7.7)	4 (66.7)	1 (1.7)
Coughing up blood	0 (0.0)	0 (0.0)	0 (0.0)
Difficulty breathing	6 (9.2)	5 (83.3)	1 (1.7)
Skin bumps or sores	2 (3.1)	2 (33.3)	0 (0.0)
Lameness or limping	2 (3.1)	1 (16.7)	1 (1.7)
Fever	5 (7.7)	4 (66.7)	1 (1.7)
Seizures	0 (0.0)	0 (0.0)	0 (0.0)
Eye or vision problems	1 (1.5)	1 (16.7)	0 (0.0)
Lethargy	8 (12.3	6 (100.0)	2 (3.4)
Poor appetite	8 (12.3)	6 (100.0)	2 (3.4)
Weight loss	3 (4.6)	2 (33.3)	1 (1.7)

*Values are no. (%) except as indicated. Clinical canine cases were defined as dogs with clinically compatible signs of blastomycosis and diagnosed by a veterinarian or having detectable *Blastomyces* antibody.
†Four dogs are missing information about time spent living in the neighborhood.
‡Distance to construction is measured as the distance in meters from the house to the nearest lot with a construction permit in 2021.
§Four dogs are missing information on age.
¶Two dogs are missing information on breed.
#Two dogs are missing information on weight.

Appendix 2 Table 3. Characteristics of households in riverside neighborhood with and without construction during 2021 during investigation of Blastomycosis cluster, Wisconsin, 2021–2022*

Characteristic	Total, n = 122	Construction during 2021, n = 18	No construction, n = 104
Years lived in neighborhood	4.9 (3.2–15.5)	0.67(0.38–4.5)	5.2 (3.5–16.3)
Median distance to river, meters (IQR)	344 (209–481)	111 (60–387)	351 (256–484)
Antibody testing	46 (37.7)	6 (33.3)	40 (38.4)
No. included	46	6	40
No. households with any antibodies detected	34 (73.9)	5 (83.3)	29 (72.5)
No. households tested with humans with detected antibodies†	31 (67.4)	5 (83.3)	26 (65.0)
No. households with canines with detected antibodies	7 (15.2)	0 (0.0)	7 (17.5)
No. households with both humans and canines with detected antibodies	4 (8.7)	0 (0.0)	4 (10.0)

*Values are no. (%) except as indicated. Antibody testing was completed by MiraVista Diagnostics for *Blastomyces* human and canine enzyme immunoassay (EIA).
†For humans, antibodies are considered detected in those returning a positive or intermediate result.

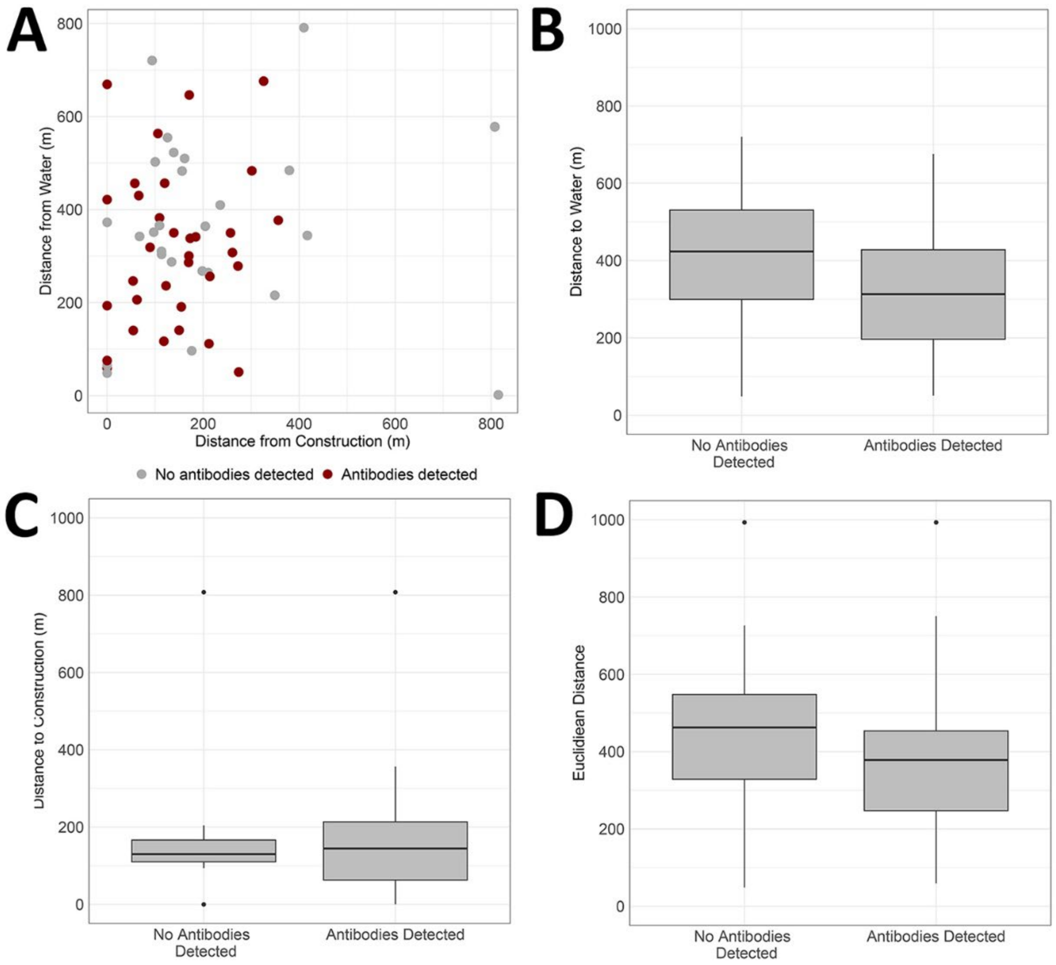

Appendix 2 Figure. Distance from house to nearest home under construction and in-neighborhood water by antibody detection during investigation of cluster of blastomycosis, Wisconsin, 2021–2022. Antibodies are marked as detected if at least one person or dog in the house had detected antibodies. For people this includes positive or intermediate results. Only houses where a household member was tested for

antibodies (n = 46) were included. A) Distance in meters from construction and water by antibody status ($p = 0.17$). B) Side by side boxplots showing distance from in-neighborhood water (meters) by antibody status ($p = 0.3$). C) Side by side boxplots showing distance from nearest home with construction (meters) in 2021 by antibody status ($p = 0.44$). D) Side by side boxplots showing Euclidean distance from in-neighborhood water and nearest home with construction in 2021 by antibody status ($p = 0.17$).
